# Supplementary figures and images for: The Impact of Smoking-Associated Genetic Variants on Post-Exercise Heart Rate
Source: Int J Mol Sci. 2025 Sep 9;26(18):8787. doi: 10.3390/ijms26188787 (PMC12469723; doi:10.3390/ijms26188787)

# Supplementary Materials

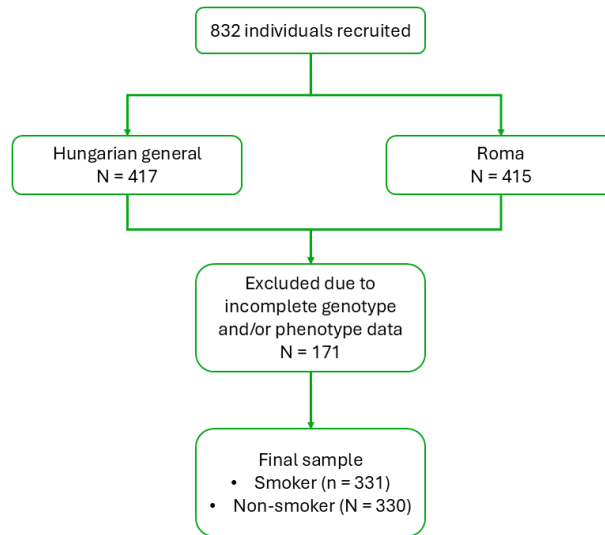

**Figure S1.** Study Participant Flowchart.

Supplement: Supplementary file 1 [file ijms-26-08787-s001.zip › ijms-3795927-supplementary.pdf]
